# A new method for evaluation of patellar height and the position of the joint line before and after total knee arthroplasty

**DOI:** 10.1186/s12891-020-03794-1

**Published:** 2020-11-21

**Authors:** Hua Han, Xiaohui Zhang

**Affiliations:** 1 Department of Orthopaedics, Lanzhou University Second Hospital, Lanzhou University, No. 82 Cuiyingmen, Chengguan District, Lanzhou City, Gansu Province 730030 China; 2 Orthopaedics 3D-Print Engineering Research Center of Gansu province, Lanzhou City, Gansu Province 730030 China

**Keywords:** Total knee arthroplasty, Patellar height, Patella baja, Joint line position, Reliability

## Abstract

**Background:**

The measurement of patellar height and restoration of the natural position of the joint line are crucial to total knee arthroplasty (TKA). However, there remains a lack of consensus on an optimal measurement method to associate the patellar height with the joint line position. The objective of this study was to introduce a new method and validate the application in TKA both preoperatively and postoperatively.

**Methods:**

Instead of taking marginal landmarks as the tibial references, the tibial shaft axis was used to construct the new measurement method, which comprises the axis-patella (AP), joint axis-patella (jAP) indices and joint line height (JLH). Patellar heights were measured using the Insall-Salvati (IS), modified Insall-Salvati (mIS), Blackburne-Peel (BP), Caton-Deschamps (CD) indices, and the new method in 175 knees both preoperatively and postoperatively. Intraclass correlation coefficients and Pearson’s correlation analyses were respectively used to evaluate the reliabilities and correlations.

**Results:**

There were good correlations between the proposed method and the mIS, CD, and BP indices. High inter-observer reproducibility was found for AP (preoperative and postoperative 0.83), jAP (preoperative 0.82; postoperative 0.86) indices and JLH (preoperative 0.88; postoperative 0.95). High intra-observer repeatability was also found for AP (preoperative 0.85; postoperative 0.87), jAP (preoperative 0.83; postoperative 0.87) indices and JLH (preoperative 0.80; postoperative 0.92).

**Conclusions:**

The new method is reliable for measuring patellar height before and after TKA, providing an alternative to distinguish between true and pseudo patella baja. Furthermore, JLH can be applied to assess and restore the joint line position in TKA.

## Background

Total knee arthroplasty (TKA) is the gold standard for the treatment of advanced knee osteoarthritis [1, 2]. However, among the postoperative complications of TKA, patellofemoral pain, knee instability, and reduction of movement range are still common, which resulted from abnormal patellar height to some extent [3, 4]. Patella alta or patella baja is considered to be one of the indicators of poor clinical outcomes after TKA. The former is associated with patellofemoral instability [5], and the latter is associated with the reduction of movement range and anterior knee pain [6, 7]. Furthermore, patellofemoral symptoms account for a large percentage of TKA revisions [6, 8]. Especially the patella baja has a high incidence after TKA, which was reported to be between 10 and 82% [2, 9, 10]. The occurrence of patellar baja after TKA depends on a shortening of the patellar tendon or an elevation of the joint line. An elevated joint line is responsible for the pseudo patella baja, whereas a shortened patellar tendon is the main cause of the true patella baja [11, 12].

The measurement of patellar height and the distinction between true and pseudo patella baja are crucial for postoperative knee dysfunction, which can search for the cause and guide the treatment [12]. Currently, the Insall-Salvati (IS) [13], modified Insall-Salvati (mIS) [14], Blackburne-Peel (BP) [15] and Caton-Deschamps (CD) indices [16] are widely used for measuring patellar height. In the context of TKA, previous reports have divided the above methods into two types [12], IS and mIS indices referencing off the insertion site of the patellar tendon on tibia, and BP and CD indices referencing off the joint line of proximal tibia. Preoperatively, severe osteoarthritis with destruction of the articular surface and marginal osteophytes formation leads to actually measurement difficulties to some extent, which are the common shortcomings of these four methods of using a marginal reference, especially when osteoarthritis is combined with severe malalignment [17]. Postoperatively, for IS and mIS indices, only when true patella baja occurs will their value be abnormally low, while the pseudo patella baja may not be detected. For BP and CD indices, a low value will be exhibited both true patella baja and pseudo patella baja. Surgeons must combine both types of indices to distinguish between true patella baja and pseudo patella baja [6, 12]. For CD index, a shift of the tibial landmarks in the anteroposterior and superoinferior direction could occur postoperatively, which is susceptible to implant design and bone resection. For BP index, a problem with postoperative measurements is the vertical line emanating from the tibial plateau line is significantly shortened evenly unavailable if the vertical line cuts the patella when a tibial component positioning with an excessive posterior inclination [17].

As mentioned above, an elevation in position of the joint line directly influences the diagnosis of pseudo patella baja. Furthermore, successful restoration of the natural position of the joint line after TKA remains technically challenging for surgeons [18]. An elevation of 1 mm in joint line has been commonly found to occur after primary TKA [18, 19]. To date, numerous empirical methods, “two finger breadths from the tibial tubercle”, and measurement techniques involving absolute values, “10 mm from the fibular styloid”, have been described to guide the restoration of the joint line during TKA [19–21]. However, there remains a lack of consensus on an optimal measurement method to associate the patellar height with the joint line position.

In view of the close relationship between patella height and joint line change in TKA, a new measurement method was proposed in this study, which can simultaneously reflect the precise change of joint line based on the determination of patella height, so that it can be easily applied to preoperative planning and postoperative evaluation. The main objective of this study was to introduce this new method and validate the application in TKA both preoperatively and postoperatively.

## Methods

### Patients selection

After approval by the institutional ethics committee, a retrospective evaluation was performed prospectively on the lateral radiographs of 369 consecutive knees of patients who had undergone a primary TKA (NexGen LPS-flex, Zimmer, Warsaw, Indiana) in our hospital between January 2018 and January 2019. All surgeries were performed by a single senior joint surgeon under subarachnoid anesthesia. And the surgical approach, patellar eversion, osteotomy and prosthetic placement were carried out in the same way. We only included patients who underwent TKA without patellar resurfacing for primary osteoarthritis. Importantly, the lateral radiographs of the involved knee were taken twice in our hospital for each patient, and the typical examination times were within 1 month before TKA and 2 weeks after TKA. In view of the fact that the following factors may have an impact on the measurement of patellar height, the exclusion criteria were determined: (1) patients who presented preoperative or postoperative radiographs with the knee in less than 30° of flexion; (2) patients who had undergone a unicompartmental knee arthroplasty (UKA), high tibial osteotomy (HTO) or a revision procedure; (3) patients with an excessively rotated knee radiographs due to poor technique, impairing an accurate identification for bone landmarks during measurement of patellar height.

Of a total of 369 knees, one hundred and seventeen (31.7%) were excluded because the preoperative lateral radiographs were taken in other hospitals; fifty-eight (15.7%) were excluded because the radiographs showing the knee in less than 30° of flexion; nine (2.4%) were excluded due to previous UKA or HTO; ten (2.7%) were excluded because the radiographic examination time did not accord with schedule. Eventually, 175 knees (corresponding to 350 lateral radiographs) of 167 patients were included in this study. Of these, eight patients had undergone bilateral TKA at different times.

### Study protocol

Using the ST-PACS CD-Medical software (version 1.5.3, Crealife, Beijing, China), the preoperative and postoperative patellar height were respectively measured on the lateral knee radiographs for each case. The IS, mIS, CD, BP indices, and the new method were applied to assessing patellar height. The measurement processes were performed independently by two trained observers in a double-blinded manner. All of the radiographs were measured by observer 1. To assess the reliability and variation in measuring patellar height, intra-observer and inter-observer studies were carried out. Two hundred radiographs of 100 knees were measured by observer 1 with an interval of 3 months, which were randomly selected from the total of 175 knees. Moreover, two hundred and twenty radiographs of 110 knees were independently measured by observer 2, which were also randomly selected from all cases.

### Description of the methods for assessing patellar height

**Fig. 1:**
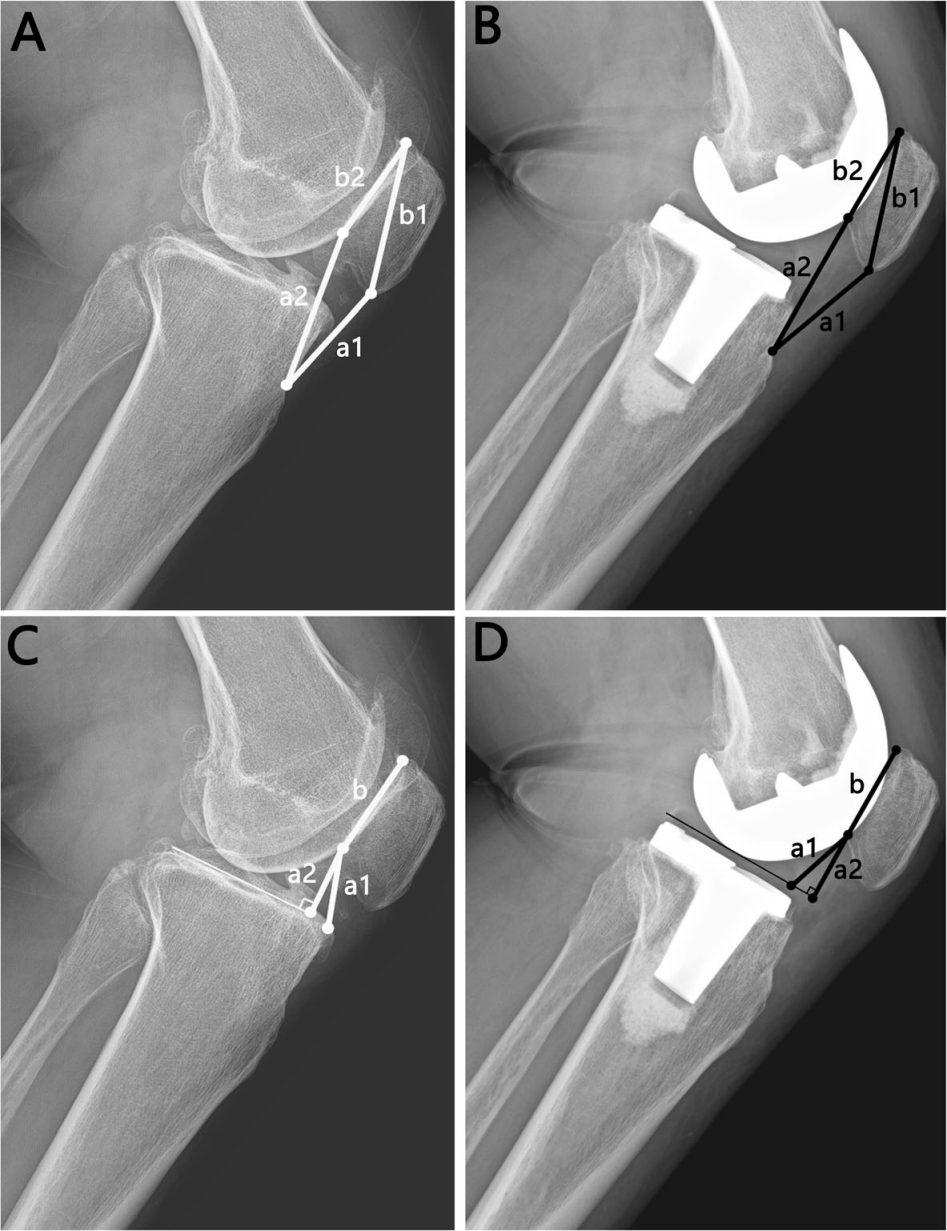
Representation of the four classical methods used for measuring patellar height. As represented in A and B (preoperative and postoperative radiographs, respectively), the Insall-Salvati index is calculated by “a1/b1”, and the modified Insall-Salvati index is calculated by “a2/b2”. As represented in C and D (preoperative and postoperative radiographs, respectively), the Caton-Deschamps index is defined as “a1/b”, and the Blackburne-Peel index is defined as “a2/b”

According to the description of the original publication [13–16], four classical indices for measuring patellar height were performed (Fig. 1A and C). Each index was drived from two measurements (a/b), “a” was the distance from the lower end of the patella to the tibial landmark and “b” was the length of the patella. For IS index, “a” was the length of the patellar tendon, which was measured from the lower pole of the patella to the insertion site of the patellar tendon on tibia (a notch superior tibial tubercle), and “b” was measured between the upper and lower pole of patella. For mIS, CD and BP indices, the length of patellar articular surface was shared as the measurement “b”. The “a” was the distance between the inferior pole of the patellar articular surface to the tibial landmarks of the three indices, including the insertion site of the patellar tendon on tibia, the antero-superior margin of the tibia, and the perpendicular intersection of the inferior pole of the patellar articular surface with the tibial plateau line, respectively. Osteophytes were excluded during the measurements. After TKA, the tibial plateau was defined as the tangent line of the distal femoral component parallel to tibial osteotomy plane, as conducted by Rogers [6] and Cabral et al. [3] (Fig. 1B and D).

### Description of the new method

In current study, a new method was proposed to evaluate the patellar height and quantitatively measure the joint line position on the lateral radiograph of the knee before and after TKA. Instead of taking marginal landmarks as the tibial references in classical methods, the tibial shaft axis was used to construct the new measurement method, which comprises the axis-patella (AP), joint axis-patella (jAP) indices and the joint line height (JLH).

**Fig. 2:**
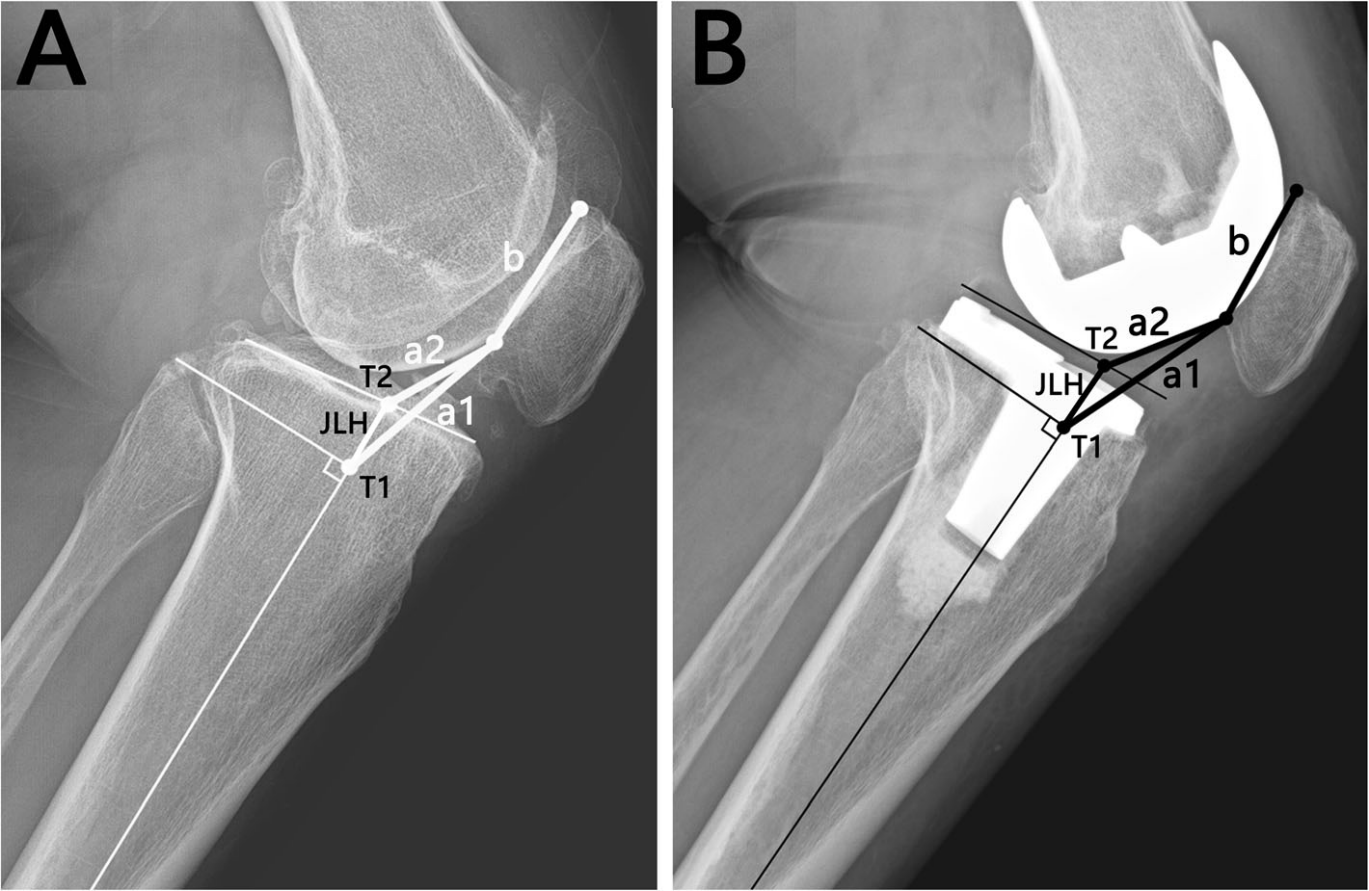
Illustration of the new method proposed in this study. The axis-patella (AP) index is defined as “a1/b”. Measurement “a1” is measured from the lower pole of the patellar articular surface to the intersection point (T1) between the tibial shaft axis and its perpendicular line passing through the tip of the fibular head. Measurement “b” is the length of the patellar articular surface. The joint axis-patella (jAP) index is calculated by “a2/b”. Measurement “a2” is measured from the lower pole of the patellar articular surface to the intersection point (T2) between the tibial shaft axis and the joint line. The joint line height (JLH) is defined as the distance between the T1 and T2

Firstly, the AP index is a ratio between two measurements (a1/b) (Fig. 2). Measurement “b” is the length of the patellar articular surface. Measurement “a1” is the distance from the lower pole of the patellar articular surface to the intersection point (T1) between the tibial shaft axis and its perpendicular line passing through the tip of the fibular head. The tibial shaft axis is the mid-diaphyseal line, as described by previous studies [22, 23]. Secondly, the jAP index is another relative index with a2/b (Fig. 2A). Measurement “a2” is the distance from the lower pole of the patellar articular surface to the intersection point (T2) between the tibial shaft axis and the joint line. The joint line was defined as the tibial plateau line preoperatively and the tangent line of the distal femoral component parallel to tibial osteotomy plane postoperatively (Fig. 2B). Thirdly, the JLH is determined by measuring the distance between the T1 and T2 on the tibial shaft axis (Fig. 2).

To investigate whether the posterior tibial slope (PTS) plays a role as a confounding factor in patellar height measurements, the PTS was measured in all cases.

### Statistical analysis

All data were collected and collated through Excel (version 2016, Microsoft, Redmond, Washington), and data analyses were performed through SPSS (version 25.0, SPSS Inc., Chicago, IL, USA). The analysis results were expressed as mean ± standard deviation. A descriptive analysis and a paired sample T test were performed to depict the distribution and difference of measurements for both pre- and post-operative knee radiographs. To verify the validity of the new method, we conducted a Pearson’s correlation analysis on the values of various measurement methods included in this study. In addition, the intraclass correlation coefficient (ICC) and Bland-Altman plots, constructed by the Medcalc software (version 11.4, MedCalc software Ltd., Acacialaan 22, 8400 Ostend, Belgium), were used to analyze the intra- and inter-observer reliability and variability [24]. *P* < 0.05 was regarded as statistically significant.

## Results

The mean age of patients at surgery was 64.22 years, and 79.6% were women (Table 1). The number of left knees (54.3%) was slightly more than right knees (45.7%). There were no significant difference between pre-operation and post-operation for IS, mIS, and AP indices. The mean CD, BP, and jAP indices were significantly lower in post-operation than pre-operation (*P* < 0.001; Table 1). Furthermore, JLH was found to be significantly greater after surgery than before surgery (*P* < 0.001), with an average increase of 3.17 ± 3.06 mm.

**Table 1:** Demographics of all subjects and baseline data for measurement parameters, before and after total knee arthroplasty

	Frequency-n (%)	Mean ± standard deviation		*P* value
		Pre-operation	Post-operation	
Patients number	167			
Males	34 (20.4)			
Females	133 (79.6)			
Knees number	175			
Left	95 (54.3)			
Right	80 (45.7)			
Age at surgery		64.22 ± 6.52 (years)		
IS		0.99 ± 0.15	0.99 ± 0.16	0.638
mIS		1.53 ± 0.17	1.52 ± 0.17	0.636
CD		0.86 ± 0.12	0.74 ± 0.13	0.000
BP		0.79 ± 0.13	0.72 ± 0.13	0.000
AP		1.34 ± 0.17	1.35 ± 0.16	0.214
jAP		0.99 ± 0.14	0.93 ± 0.14	0.000
JLH		10.89 ± 3.12	14.06 ± 3.07	0.000
PTS		9.09 ± 3.53	8.97 ± 2.43	0.684
JLH (post-pre)		3.17 ± 3.06 (mm)		

*IS* Insall-Salvati, *mIS* Modified Insall-Salvati, *CD* Caton-Deschamps, *BP* Blackburne-Peel, *AP* Axis-patella, *jAP* Joint axis-patella, *JLH* Height of the joint line, *PTS* Posterior tibial slope, *JLH (post-pre)* difference of JLH between post-operation and pre-operation

As shown in Table 2, good correlations were found between AP index and mIS, CD, and BP indices both preoperatively (0.62 to 0.70) and postoperatively (0.65 to 0.76). The jAP index also has a good correlation with the above three classical indices before (0.60 to 0.78) and after surgery (0.52 to 0.79), it has the highest correlation with CD index and was higher postoperatively than preoperatively (0.79 and 0.78, respectively). However, IS index did not present a good correlation with the other indices. In addition, poor correlations were found between the PTS and the various indices of patellar height, further details are presented in Table 2.

**Table 2:** Pearson’s correlation coefficients among measurement parameters, before and after total knee arthroplasty

Measurement indices	Pre-operation							Post-operation						
	JLH	AP	jAP	IS	mIS	CD	BP	JLH	AP	jAP	IS	mIS	CD	BP
PTS	0.28	−0.11	−0.32	−0.02	−0.19	−0.28	−0.39	0.17	−0.03	−0.11	−0.04	− 0.02	− 0.10	− 0.14
AP			0.76	0.19	0.69	0.70	0.62			0.66	0.25	0.76	0.68	0.65
jAP				0.27	0.60	0.78	0.70				0.21	0.52	0.79	0.74
IS					0.29	0.28	0.32					0.32	0.28	0.27
mIS						0.76	0.76						0.57	0.58
CD							0.92							0.94

*PTS* Posterior tibial slope, *AP* Axis-patella, *jAP* Joint axis-patella, *IS* Insall-Salvati, *mIS* Modified Insall-Salvati, *BP* Blackburne-Peel, *CD* Caton-Deschamps, *JLH* Height of the joint line

The inter- and intra-observer agreements of all indices both preoperatively and postoperatively were calculated by ICC, with 95% confidence intervals, are shown in Table 3. There was a high intra-observer agreement between twice measurements by observer 1 concerning all indices before (0.73 to 0.92) and after TKA (0.71 to 0.92). Among overall indices, the IS index showed the highest ICC both preoperatively (0.92) and postoperatively (0.92). We found that the AP and jAP indices presented high ICCs preoperatively (0.85 and 0.83, respectively) and postoperatively (0.87 for both indices). While high ICCs were also showed preoperatively (0.78, 0.73, and 0.79, respectively) and postoperatively (0.71, 0.87, and 0.87, respectively) concerning the mIS, CD and BP indices.

**Table 3:** Inter- and intra-observer agreement of the six methods used, before and after total knee arthroplasty

	Pre-operation							Post-operation						
	IS	mIS	CD	BP	AP	jAP	JLH	IS	mIS	CD	BP	AP	jAP	JLH
Intra-observer agreement														
ICC	0.92	0.78	0.73	0.79	0.85	0.83	0.80	0.92	0.71	0.87	0.87	0.87	0.87	0.92
95% confidence interval	0.87–0.95	0.68–0.84	0.63–0.81	0.70–0.86	0.78–0.89	0.75–0.88	0.71–0.86	0.88–0.94	0.60–0.75	0.81–0.91	0.81–0.91	0.80–0.91	0.80–0.91	0.89–0.95
*P* value	0.000	0.000	0.000	0.000	0.000	0.000	0.000	0.000	0.000	0.000	0.000	0.000	0.000	0.000
Sample size	100	100	100	100	100	100	100	100	100	100	100	100	100	100
Inter-observer agreement														
ICC	0.85	0.69	0.68	0.81	0.83	0.82	0.88	0.86	0.54	0.86	0.83	0.83	0.86	0.95
95% confidence interval	0.79–0.90	0.58–0.78	0.56–0.77	0.73–0.87	0.75–0.88	0.74–0.87	0.82–0.91	0.76–0.91	0.37–0.67	0.81–0.90	0.76–0.88	0.76–0.88	0.80–0.90	0.93–0.97
*P* value	0.000	0.000	0.000	0.000	0.000	0.000	0.000	0.000	0.000	0.000	0.000	0.000	0.000	0.000
Sample size	110	110	110	110	110	110	110	110	110	110	110	110	110	110

*ICC* Intraclass correlation coefficient, *IS* Insall-Salvati, *mIS* Modified Insall-Salvati, *BP* Blackburne-Peel, *CD* Caton-Deschamps, *AP* Axis-patella, *jAP* Joint axis-patella, *JLH* Height of the joint line

There was a high inter-observer agreement of all indices between two observers (preoperative 0.68 to 0.85, postoperative 0.54 to 0.86). Of these methods, the ICCs of AP and jAP indices respectively were 0.83 and 0.82 preoperatively, 0.83 and 0.86 postoperatively, showing a high inter-observer reproducibility.

**Fig. 3:**
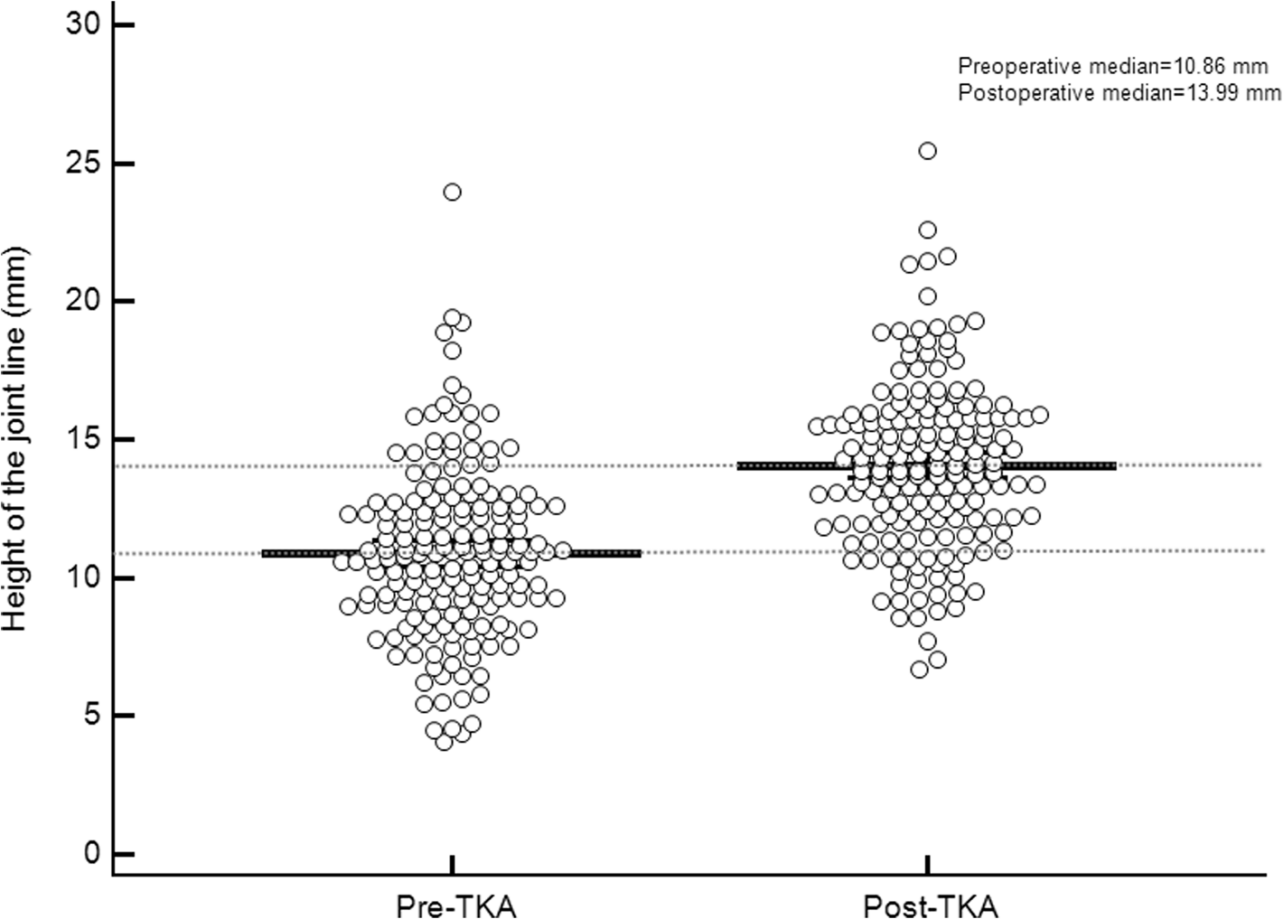
The scatter chart exhibiting the change trend of the joint line height in 175 cases both preoperatively and postoperatively

It is worth mentioning that JLH showed an excellent intra-observer repeatability (preoperative 0.80, postoperative 0.92) and inter-observer reproducibility (preoperative 0.88, postoperative 0.95). We used a scatter chart to demonstrate the change trend of JLH in 175 TKA procedures (Fig. 3). Each point represented an absolute value of JLH. It can be clearly found that the median was higher after TKA (13.99 mm) than before TKA (10.86 mm).

**Fig. 4:**
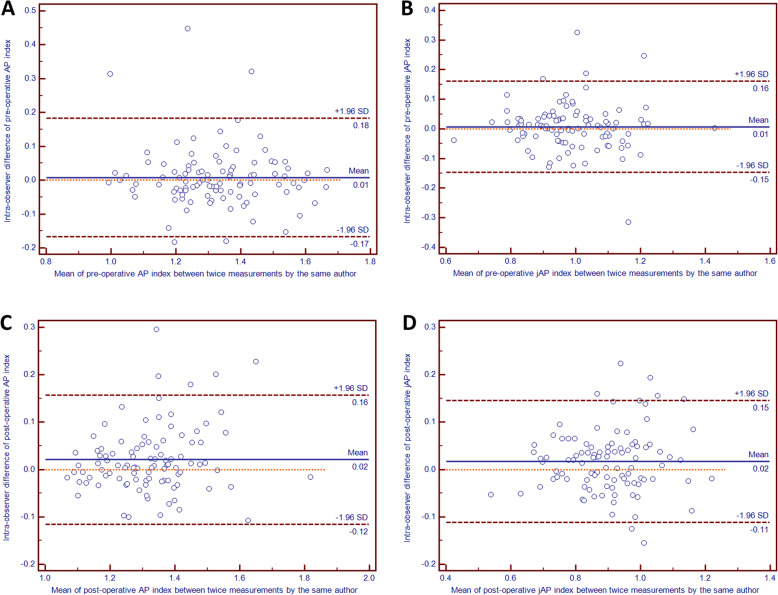
Bland-Altman plots of the preoperative and postoperative differences for measuring patellar height between twice measurements by the same author with a 3 months interval: the axis-patella (AP) index (**A** and **C**, respectively), the joint axis-patella (jAP) index (**B** and **D**, respectively)

**Fig. 5:**
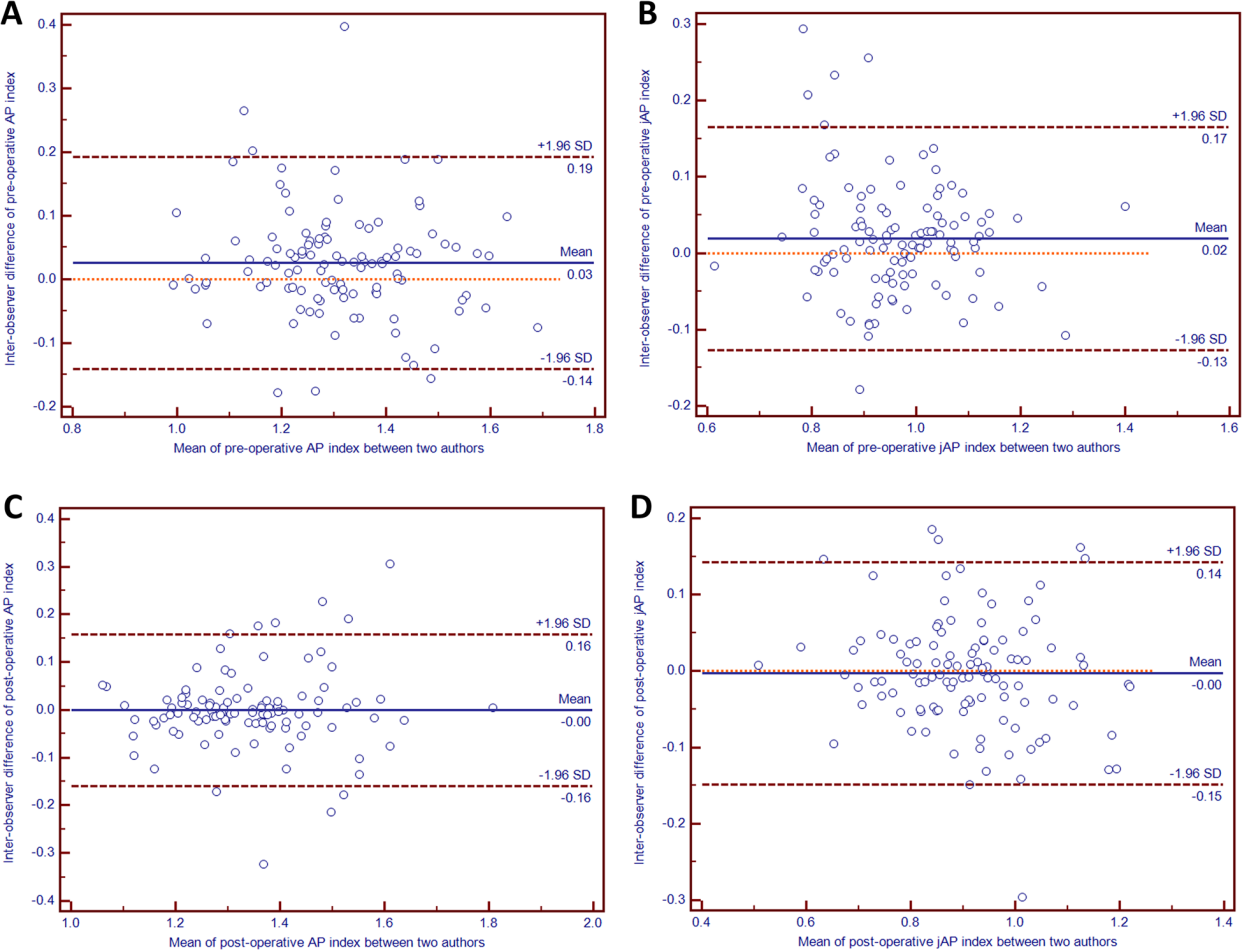
Bland-Altman plots of the preoperative and postoperative differences for measuring patellar height between two authors: the axis-patella (AP) index (**A** and **C**, respectively), the joint axis-patella (jAP) index (**B** and **D**, respectively)

For AP and jAP indices, the Bland–Altman plots with 95% limit of agreements depicted revealed not only good intra- and inter-observer agreement but also differences being close to zero with no evidence of systematic biases (Fig. 4 and Fig. 5).

## Discussion

The primary purpose of this study was to introduce a new method for measuring patellar height and assess the validity by comparing with the four commonly used methods. The results indicated that a high inter- and intra-observer reliability was found for AP and jAP indices. Pearson’s correlation analysis showed that the AP and jAP indices were strongly correlated with the four classical counterparts. The new method proposed has been demonstrated of capable to evaluate patellar height both preoperatively and postoperatively for patients undergoing TKA due to primary osteoarthritis.

All four of classical methods mentioned in this article have intrinstic methodological difficulties, especially in patients who performed TKA resulted from severe osteoarthritis, which may lead to an increase in inter- and intra-observer variability [25–28], and evenly the measurement may be unavailable. Preoperatively, severe osteoarthritis with destruction of the articular surface and osteophytes formation, especially combined with lower extremity malalignment, usually leads to measurement errors to some extent, which are the common shortcomings of these four methods of using a marginal reference [17]. For IS and mIS indices, it is imperative to standardize the tibial attachment of the patellar tendon in order to avoid a significant variation due to the obscure morphology and the pathological overgrowth of the tibial tubercle [26, 29]. Besides, the variants of patellar shape can bring about a greatly different result for IS index, especially in a patella being characterized by a long, non-articulating inferior pole (Cyrano patella) [12, 28, 29]. For BP index, one problem is the determination of the tibial plateau line after TKA. A joint line is constructed on the tibial articular surface, which is difficult to determine each time postoperatively due to the variation of polyethylene insert. As described by Rogers et al. [6], the mean values of tibial insert thickness had to be used and compensate for the shape of the polyethylene insert, whereas the use of the mean value is often not an accurate substitute for individual prosthetic height in clinical measurements, in which requires knowing the thickness of the insert. On the other hand, the tibial component is usually placed with a posterior slope, but the vertical line (Fig. 1d) emanating from the tibial plateau line is significantly shortened evenly unavailable if the vertical line cuts the patella when a tibial component positioning with an excessive posterior inclination [17], although PTS showed a poor correlation with BP in this study. For CD index, preoperatively, it is located around a point outside the joint line, both in anterior and superior direction, the tibial reference point is difficult to determine particularly in severe arthritic knee due to osteophyte attachments. Postoperatively, however, the tibial component become a part of the articular surface, which is more precisely formed by the insert. This shows a shift of the reference point in the anteroposterior and superoinferior direction owing to thickness of bone resection and implant design. Thus, the comparability of the pre- and postoperative reference point is questionable [17, 25]. In view of this, it is necessary to find an accurate measurement method, in which the reference point is relatively constant and comparable before and after surgery.

The tibial landmark of the AP index, formed by the tibial shaft axis and its perpendicular line passing through the tip of the fibular head, intrinsically owns better positional stability before and after TKA. Compared with the tibial plateau, the fibular head is independent of TKA, as well as osteophytes or the destruction of tibiofemoral articular surface. And the recognition of the styloid process of fibular head is accurately acquired on the standard lateral radiograph [20, 30]. In this study, the tibial shaft axis was determined by selecting two mid-diaphyseal points at 70- and 110-mm distal to the tibial plateau down the tibial shaft, in which could avoid an increase of the measurement variability, as described by previous studies [22, 23]. As an intramedullary reference, the tibial shaft axis can theoretically provide a constant landmark compared to marginal counterparts, especially during tibial rotating subsequent to knee flexion. In present work, no significant difference was found between pre-operation and post-operative 2 weeks for AP and IS indices, consistently with the short-term results of IS index in previous reports [3, 31]. Hence, we speculate that this result is reasonable, because the T1 point on the tibial shaft axis, which located at the level of the tip of the fibular head, maintains a relatively constant distance from the tibiofemoral surface affected by the surgical operation. It may attribute to the constant characteristics of the T1 point, in the both anteroposterior and superoinferior direction, which is highly similar to the attachment point of the patellar tendon on the tibia in the IS index.

Previous studies have proved that the patella baja is a common complication of TKA [2, 9], which can be classified as a true patella baja caused by the shortening of the patellar tendon and the pseudo patella baja caused by the elevating of the joint line [12, 32]. The incidence of the pseudo patella baja following TKA had been published in 34–65% [11]. As described by Grelsamer [12], CD and BP indices using the tibial reference at joint line can be applied to determine the true patella baja and pseudo patella baja, whereas the IS and mIS indices can be used to identify patellar tendon shortening. Generally, surgeons must combined both types of methods to distinguish the true and pseudo patella baja. In this study, the jAP index was found to have a strong correlation with CD and BP indices. In addition, when comparing the pre- and post-operative values of the jAP, CD and BP indices, all three indices showed a synergistic decreasing trend of patella. The small difference in the pre- and postoperative values can certainly be explained in large part by the elevation of the joint line postoperatively. In this study, the jAP index also presented an excellent interobserver reproducibility and intraobserver repeatability. The potential explanation is that the determination of the T2 reference point is independent of the implants design. Meanwhile, the tibial shaft axis usually passes through the anterior-middle segment of the tibiofemoral joint line on the lateral radiographs, the measurement “a” of jAP index is less likely to be affected by an excessive posterior inclination than BP and CD indices. In fact, we believe that the theoretical advantage of jAP index is that the T2 point will shift correspondingly along the tibial shaft axis with the change of the joint line position postoperatively, with less error in the anteroposterior direction. Therefore, once true patella baja is diagnosed on a lateral knee radiograph of postoperative TKA, both the AP and jAP indices will be abnormally low. When pseudo patella baja occurs, the AP index remains normal, but the jAP index will decrease, correspondingly JLH will increase and can accurately reflect the change of joint line height when compared with the preoperative JLH value (Fig. 3).

Of the widely accepted surgical goals of TKA, restoration of the joint line is crucial. Accurately restoring the natural joint line is a challenge and one of the most important factors in achieving satisfied clinical outcomes [19, 20, 33]. Nevertheless, accurate restoration of the joint line depends on the anatomical landmarks of the knee [19, 20]. Typically, in complicated or revision TKA, these landmarks are commonly obscured even missing, resulting in a difficult in restoration [19, 21]. Pereira et al. [20] described a series of procedures to determine the joint line position using the following bony landmarks: tibial tubercle, proximal tibio-fibular joint, femoral epicondyles, and femoral metaphyseal flares. Laskin [21] used the fibular styloid and medial epicondyle as reference points to measure the joint line during TKA. However, there remains a lack of consensus on an optimal measurement method. Moreover, several clinical retrospective studies and biomechanics evaluations had proved that postoperative joint line elevation of more than 5 to 8 mm is closely related to poor clinical outcomes, such as anterior knee pain, reduction of movement range, knee instability, and accelerated polyethylene wear [18, 19, 33]. Therefore, there should be a robust method to accurately determine the level of bone resection, thus to reproduce the position of the natural joint line. As a vital element of the new method, the measurement of JLH provides feasibility for quantitative analysis of the alteration of the joint line on the lateral radiograph before and after TKA. In this study, the JLH averaged 10.89 mm before TKA, and 14.06 mm after TKA, with an average elevation of 3.17 mm (paired Student’s test *P* < 0.001) (Table 1).The JLH was measured directly on the tibial shaft axis, which is strongly associated with biomechanics and component positioning of prosthetic knee [34]. Furthermore, because the tibial shaft axis is determined by connecting two mid-diaphyseal points at 70- and 110-mm distal to the tibial plateau down the tibial shaft, it is less prone to intra- and interobserver variation (ICCs: 0.80–0.95). In the lateral radiographs, compared with the reference points located outside the axis, we speculate that the reference points located on the tibial axis are more direct for guiding the restoration of the joint line and further evaluation of the knee instability, especially in flexion instability.

### Limitations

There are several limitations of this study. Firstly, the retrospective nature is clear limitation of the study, and the research data were extracted from the hospital information system. Secondly, a normal reference range was not provided for the new indices considering the included cases suffering from severe osteoarthritis. Thirdly, although the osteophytes were excluded out during measurement preoperatively and removed intraoperatively, they could remain a source of error. Finally, the patellar articular surface in the postoperative radiographs was occasionally not completely assessable, since it was partially embedded in the femoral component and therefore had to be estimated. Despite limitations, there are many strengths like use of the same prosthesis, a single senior joint surgeon being the main operating surgeon, in an effort to minimize variation.

## Conclusions

In summary, the new method comprising the AP, jAP indices and JLH is reliable for measuring patellar height before and after TKA. There are comparable inter-observer reproducibility and intra-repeatability with the commonly used methods. The application of this method provides an alternative to distinguish between true patella baja and pseudo patella baja. Furthermore, JLH can be applied broadly to quantitatively analyze the joint line position for intraoperative restoration and postoperative evaluation of TKA on the lateral radiographs.

## Data Availability

The data and materials in current paper may be made available upon request through sending e-mail to first author.

## Abbreviations

TKA: total knee arthroplasty
IS: Insall-Salvati
mIS: modified Insall-Salvati
BP: Blackburne-Peel
CD: Caton-Deschamps
UKA: unicompartmental knee arthroplasty
HTO: high tibial osteotomy
AP: axis-patella
jAP: joint axis-patella
JLH: joint line height
PTS: posterior tibial slope
ICCs: intraclass correlation coefficients
